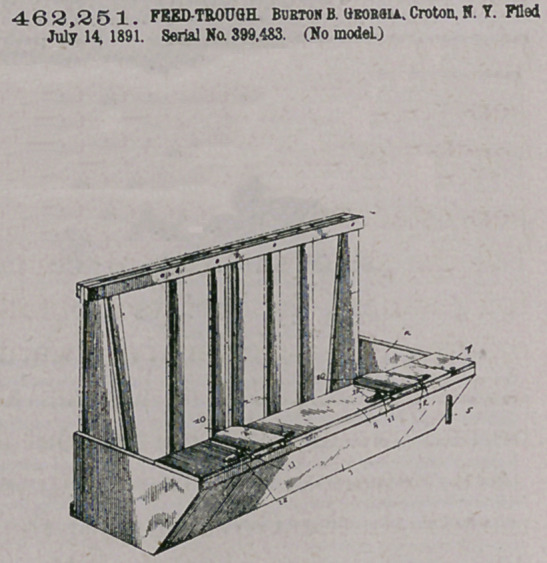# Recent Patents

**Published:** 1892-01

**Authors:** 


### RECENT PATENTS

### RELATING TO

### VETERINARY MEDICINE AND ANIMAL INDTSTRY

*Issued by U. S. Patent Office since August, 1891.*

**461,733.** HORSESHOE. JOSEPH BRÔONETT, Brooklyn, N. Y. Filed May 18, 1891. Serial No. 393,120. (No model.)

*Claim.*—1. A horseshoe comprising two similar parts hinged together at the toe, shields secured to and projecting from the parts of the shoe and hinged together at the front, bands secured to the upper edges of the shields, and a clamping-bolt for connecting the ends of the bands, substantially as described.

2. A horseshoe consisting of two similar parts hinged together at the toe, shields 12, secured to the parts of the shoe and provided at the front with interlocking knuckles 13, through which pass the pintle 13ᵃ, the bands 14, extending around the upper edge of the shields and provided with the flanges 15, one of which is screw-threaded, and the clamping-bolt 16, substantially as herein shown and described.

**461,958.** FASTENING FOR HORSE-BLANKETS. MORRIS T. MARTIN, Lake Villa, assignor to William Shakel, Chicago, Ill. Filed Apr. 1, 1891. Serial No. 387,211. (No model.)

*Claim.*—A horse-blanket fastening consisting of the straps E E, secured to the blanket, and the removable elastic section A, having facings C on each end, and each face carrying buckles for the straps E E, substantially as described.

**462,664.** UDDER-PROTECTOR. HENRY H. CHASE, Geneseo, Ill. Filed Feb. 11, 1890. Serial No. 340.076. (No model.)

*Claim.*—In an udder-protector, in combination, the udder-supporting bag having a V-shaped formation at its upper edge and conforming below said edge substantially to the cow's bag, being closed at its rear and front and provided with a buckle at each of its front upper ends and with a ring at its rear central upper end, the extensible surcingle B, adapted to be passed entirely around the body of the cow immediately forward of her hind legs, two cow-bag take-up buckling-straps E, attached to the surcingle and connecting with the buckles of the front ends of the udder-protecting bag, said surcingle also having an upper central backwardly-extending buckling portion D, and the two rear supporting-straps C C', connected to the ring c' of the rear central end of the protector-bag and to the backwardly-extended portion D of the surcingle, the whole forming an udder-protector which, while it is closed at both ends, is adjustable and can be readily manipulated for milking purposes and as readily fastened up in position around the cow's bag, substantially as described.

**461,957.** HORSE-BLINDER. GEORGE J. KANNMACHER, Rockford, Ill. Filed Mar. 12, 1891. Serial No. 384,832. (No model.)

*Claim.*—A horse-blinder comprising a frame located between the horse's ears and eyes and secured to the brow-band, said frame covered with leather and having a slot along its lower edge, a spring-actuated roller supported by the frame, a curtain of flexible material connected with the roller and extending through the slot, an ornamental stiffening-piece secured to the lower edge of the curtain of sufficient dimensions to cover the horse's eyes when drawn down, and a flexible connection between the stiffening-piece and the driver for drawing the curtain down over the horse's eyes against the action of the spring.

**461,419.** HORSE-COLLAR. EDWIN L. BRUNDAGE, East Orange N. J. Filed July 14, 1890. Serial No. 358,769. (No model.)

*Claim.*—1. A horse-collar comprising the body portion and the yielding spring-cushion, and the said cushion comprising hoop-springs secured to the body portion thereof and having a suitable covering, substantially as and for the purpose described.

2. A horse-collar comprising hoop-springs split and their meeting ends secured to the frame or body part at the front and having a suitable covering, substantially as and for the purpose described.

3. A horse-collar comprising the body portion and the yielding spring-cushion, the said cushion comprising hoop-springs secured to the body portion thereof and having a suitable covering combined therewith, the draft-eyes E, and extension *d d*, the same constituting hames, substantially as and for the purpose described.

**462,385.** VETERINARY INSTRUMENT. SANDFORD C. MEDDICK, Ovid, N. Y. Filed Nov. 6, 1890. Serial No. 370.456. (No model.)

*Claim.*—1. As an improved article of manufacture, an inflexible rubber tube formed at one end with a flaring mouth and contracted at the opposite end, said tube being reduced upon its exterior intermediate ends and having a flexible rubber tube fitting within the reduced portion and provided with oblique flanges, substantially as and for the purposes described.

2. As an improved article of manufacture, a veterinary instrument for the purpose described, consisting of a hard-rubber funnel formed with an outwardly-projecting annular flange at its mouth and with a contracted and rounded end opposite the mouth, a portion of the intervening space between said mouth and rounded end being contracted and its surface serrated or corrugated, a soft-rubber sleeve or covering corresponding and applied to said contracted portion and binding in the serrations or corrugations to retain the sleeve permanently in place, the periphery of said sleeve being flush with the adjacent portions of the funnel, and the oblique annular flanges or fins projecting from the sleeve, substantially as and for the purpose set forth.

**464,539.** OX-COLLAR. MOSS T. WHALEY, Hawley, Minn. Filed June 30, 1891. Serial No. 398,064. (No model.)

*Claim.*—In an ox-collar, the combination, with the opposite hames, the upper and lower adjustable connecting-straps, the independent pads located at the inner sides of the sections, and the L-shaped staples secured to the front faces of the hame-sections, of the neck-straddling saddle consisting of the transversely-arched metal stiffening-plates, the leather shield riveted to the under side thereof, and the curved oblong metal loop terminating at opposite sides of the saddle and the opposite adjustable strap-loops connecting the opposite ends of the oblong loop with the L-shaped staples of the hames, substantially as specified.

**461,496.** HORSE-TAIL HOLDER. ANTON DIEBOLD, Harrison, N. J. Filed June 12, 1891. Serial No. 395,973. (No model.)

*Claim.*—1. In a tail-protector, the combination, with a curved tail-plate, of a detachable ornamental plate secured to the inside of said tail-plate, two sets of strips or bands fastened to the edge of said plate and adapted to form when closed a clasp for the tail, and teeth or pins extending downward on the inside of said plate adapted to engage the hair of the tail, all said parts being adapted and arranged to operate substantially as described, and for the purposes set forth.

2. In a tail-protector, the combination, with a curved tail-plate composed of two parts pivoted together in the center, of a detachable ornamental plate secured to the inside of said tail-plate, strips or bands fastened to the edge of said plate and adapted to form when closed a clasp for the tail, and teeth or pins extending downward on the inside of said plate adapted to engage the hair of the tail, all said parts as described and set forth.

**462,649.** CATTLE-GUARD FOR RAILWAYS. FREDERICK G. BOTSFORD, Cleveland, Ohio. Filed Oct. 30, 1890. Serial No. 369,762. (No model.)

*Claim.*—The herein-described cattle-guard for railways, consisting of two sets of corrugated cross-bars secured together at their depressed portions, the apices of the bars of one set being on a higher level than those of the bars which cross them, substantially as described.

**463,069.** FEED-TROUGH. LOUIS MÜLLER, Wernigerode, Germany. Filed Apr. 28, 1891. Serial No. 390,738. (No model.)

*Claim.*—1. The combination, with the cage or closure and the sliding feeding-pan, of the sliding grid or door, and the hand-actuated rod hinged to said grid or door and the feeding-pan support, respectively, substantially as specified.

2. The combination, with the cage or closure having the guide and the feeding-pan support adapted to slide upon said guide, of the grid comprising hollow bars adapted to slide upon the bars of the cage or closure, and the hand-actuated rod hinged to the cross-piece of said grid and to said feeding-pan support, respectively, substantially as set forth.

**461,457.** TIME STOCK-FEEDER. CHARLES H. TERRY, Chicago, Ill., assignor of one-half to Theodore F. Lawrence, same place. Filed Jan. 19. 1891. Serial No. 378,277. (No model.)

*Claim.*—1. In a measuring device, the combination, with a suitable frame-work, of a spout A, secured thereto, having a discharge-valve at the bottom, a supply-spout D, also secured to said frame-work and connected with a quantity of the article to be measured, a movable spout C, telescoping at the opposite ends with the spouts A and D, respectively, a valve applied to the spout C, adapted to close the passage through the same, and means for clamping the spout C in any desired position, substantially as described.

2. The combination of a series of receptacles located at different points, each of which is provided with a discharge-valve and a suitable device for opening said valve, a connecting device uniting all of said opening devices, means for operating said opening devices at a desired point, independent locking devices applied to said valves, respectively, and independent connecting devices between each of said locking devices and the desired point, whereby all of said valves may be operated simultaneously from said point or as many as desired may be locked and the remainder simultaneously operated, substantially as described.

3. In a stock-feeding device, the combination of a series of spouts, each connected with a supply of feed and each provided with a cut-off valve and a discharge-valve, and suitable connections between the cut-off valves and the discharge-valves, respectively, whereby both series of valves may be operated from a single point, substantially as described.

4. In a stock-feeding device, the combination of a series of feed-spouts connected with a feed-supply, a series of cut-off valves applied to said spouts, respectively, suitable connections between said cut-off valves and a given point, means located at said point and adapted to operate said valves through said connections, a series of discharge-valves applied to said spouts, respectively, releasing devices applied to said discharge-valves, a connecting device uniting all of said releasing devices and leading to the given point, means located at said point for operating said releasing devices through said connecting device, a series of locking devices applied to said discharge-valves, respectively, each provided with a connecting device leading to the given point, and means located at said point for operating each of said locking devices, substantially as described.

5. The combination, with a series of receptacles A, having discharge-valves B at their bottoms, of an oscillating rod I, bearing-arms J beneath the discharge-valves, a lock L, provided with means of engagement with the rod I to secure said rod against rotation, a suspended weight M, adapted when released to disengage the lock L from the rod I, a time mechanism provided with suitable connections with the weight M to suspend said weight, and a trip applied to said time mechanism, adapted to release said weight at a predetermined moment, substantially as described.

6. The combination of a series of feed-spouts, a series of cut-off valves arranged therein, a sliding bar extending through the whole series, and a series of rods H, pivoted to the cut-off valves and hooked to the sliding bar, substantially as described.

7. The combination of a series of feed-spouts having hinged valves B at the bottoms, means for simultaneous releasing of said valves from a given point, a series of locking-levers S, adapted for engagement with said valves, a series of springs applied to said levers and adapted to operate them in one direction, and a series of connecting devices between said locking-levers and the given point, through which said locking-levers may be separately operated in the other direction, substantially as described.

**462,270.** SUTURE-INSTRUMENT. DAVID S. MCCONNAUGHEY, Washington, Iowa. Filed Mar. 30. 1891. Serial No. 387,004. (No model.)

*Claim.*—1. In a suture-instrument, the combination, with a pair of forceps having the opposing edges provided with curved recesses, of a shield having its slitted end contiguous to the said jaws.

2. In a suture-instrument, the combination of the curved recessed forcep-jaws, the slitted shield, and the cutter-provided pivoted arm, substantially as described.

3. In a suture-instrument, the combination of a pair of interpivoted forcep-jaws, a slitted shield having the same pivot as said jaws, and an arm pivoted to each of the jaws and provided with a cutting end, substantially as described.

4. The combination of the interpivoted forcep-plates having jaws $A^2$ $A^3$, recessed at *e* and *d*, the shield B, carried by pivot D, which connects the jaws, said shield having the end slit B', and having also the oppositely-projecting springs *b b* between the lever-handles, and the cutter-arm C, pivoted to each of the aforesaid handles and having the curved cutting-recesses C', all arranged substantially as described.

5. In a suture-instrument, a pair of interpivoted forcep-handles having jaws provided on their opposite inner edges with curved recesses adapted to receive a cleft-disk and compress the same tightly upon a wire.

6. The combination, in a suture-instrument, of a pair of pivoted forcep-jaws adapted to compress a disk between them and a third cutting-jaw having its arm pivoted to each of the forcep-jaws and its cutting-edge in proximity to the recessed faces of the jaws, substantially as described.

**462,251.** FEED-TROUGH. BURTON B. GEORGIA, Croton, N. Y. Filed July 14, 1891. Serial No. 399,483. (No model.)

*Claim.*—A watering-trough arranged on one side of a stanchion at the head of a feed-trough and extending longitudinally of and within the latter, said trough being provided with a top having a series of openings 9 and a pivoted cover 7, forming a continuation of the top, combined with covers 10, hinged each at one end at the head of the trough and projecting slightly beyond the top and adapted to be raised by the nose of an animal and to drop back by their own weight, a supply-pipe, an overflow-pipe, and a strainer for the overflow-pipe, substantially as described.

## Figures and Tables

**Figure f1:**
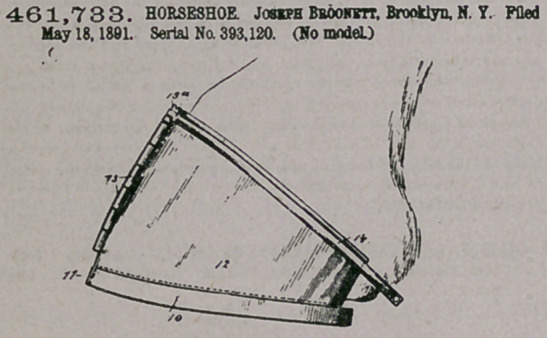


**Figure f2:**
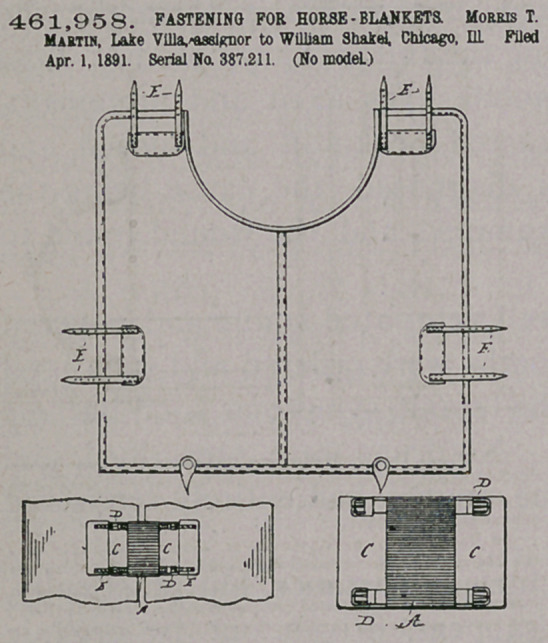


**Figure f3:**
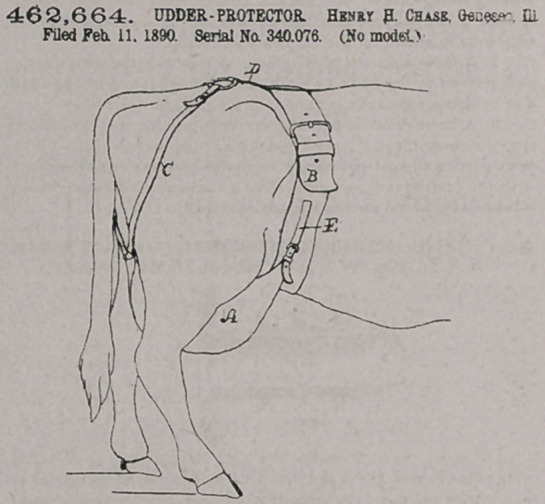


**Figure f4:**
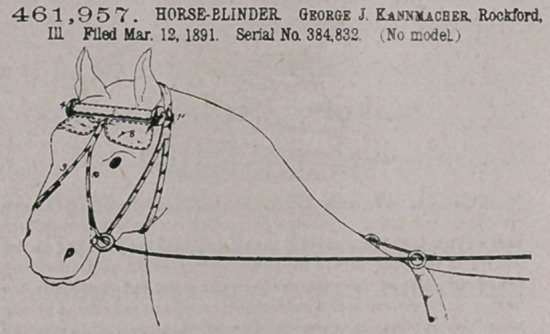


**Figure f5:**
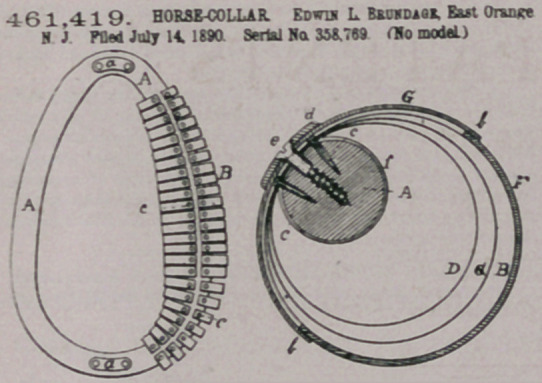


**Figure f6:**
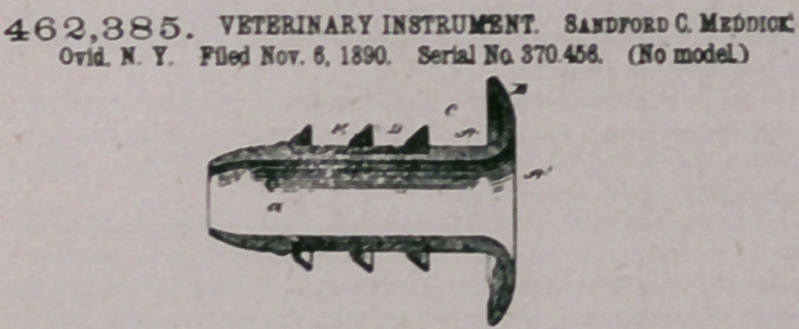


**Figure f7:**
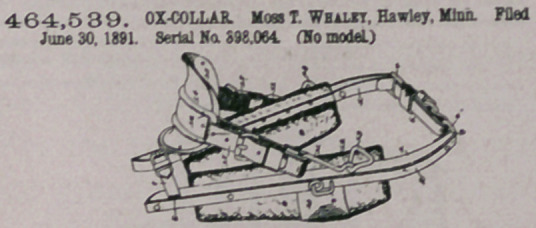


**Figure f8:**
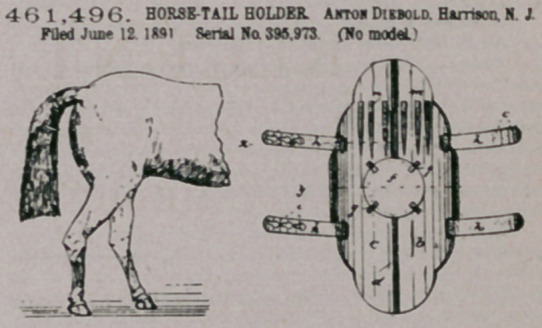


**Figure f9:**
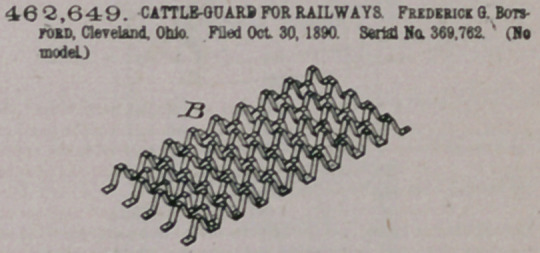


**Figure f10:**
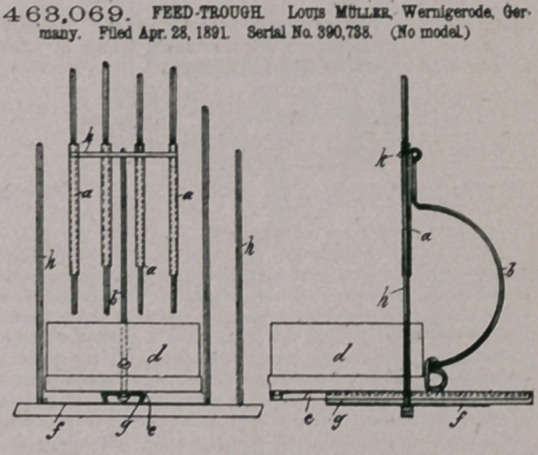


**Figure f11:**
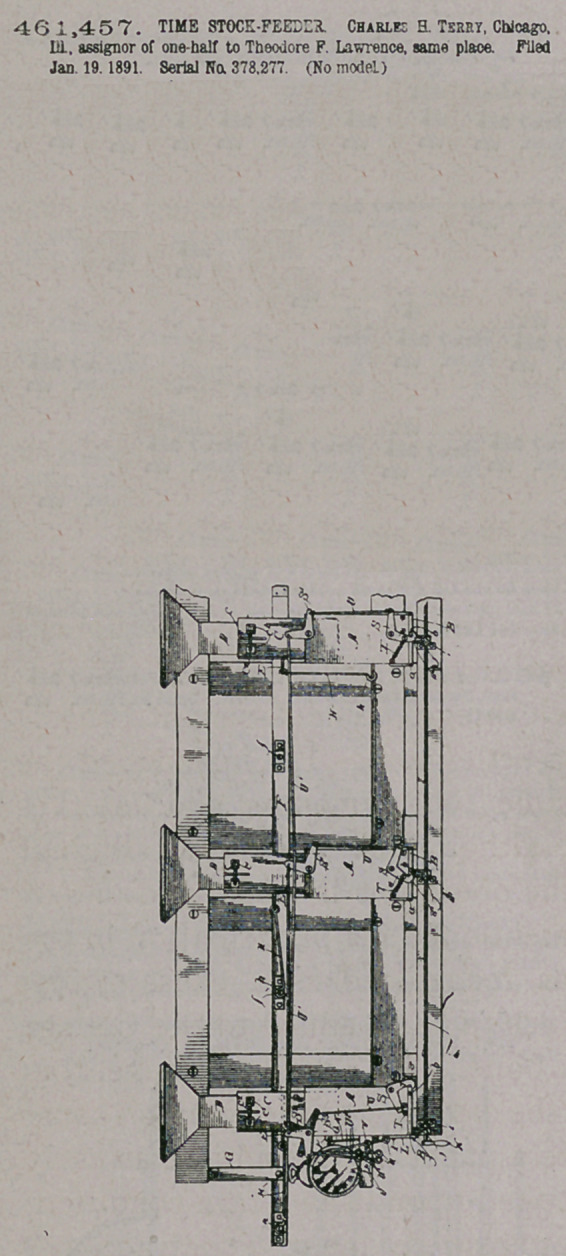


**Figure f12:**
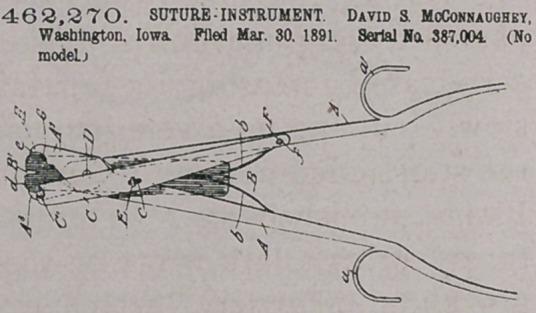


**Figure f13:**